# Distributed One Time Password Infrastructure for Linux Environments

**DOI:** 10.3390/e20050319

**Published:** 2018-04-26

**Authors:** Alberto Benito Peral, Ana Lucila Sandoval Orozco, Luis Javier García Villalba, Tai-Hoon Kim

**Affiliations:** 1 Group of Analysis, Security and Systems (GASS), Department of Software Engineering and Artificial Intelligence (DISIA), Faculty of Computer Science and Engineering, Universidad Complutense de Madrid (UCM), 28040 Madrid, Spain; 2 Department of Convergence Security, Sungshin Women’s University, 249-1 Dongseon-Dong 3-ga, Seoul 136-742, Korea

**Keywords:** distributed systems, authentication, OTP, PAM, PKI, cryptography, zero-knowledge proofs

## Abstract

Nowadays, there is a lot of critical information and services hosted on computer systems. The proper access control to these resources is essential to avoid malicious actions that could cause huge losses to home and professional users. The access control systems have evolved from the first password based systems to the modern mechanisms using smart cards, certificates, tokens, biometric systems, etc. However, when designing a system, it is necessary to take into account their particular limitations, such as connectivity, infrastructure or budget. In addition, one of the main objectives must be to ensure the system usability, but this property is usually orthogonal to the security. Thus, the use of password is still common. In this paper, we expose a new password based access control system that aims to improve password security with the minimum impact in the system usability.

## 1. Introduction

The software industry has experienced a high growth in the last decades, extending its applications to new domains, in both academic and professional fields. The use of software services to accomplish critical tasks makes it essential to provide secure access control to these services. It is common that developers offer to clients complete solutions including the hardware devices where the applications providing the required capabilities are hosted. Performing proper user and permission management in the system and providing robust authentication mechanisms avoiding non-authorized accesses are essential to ensure the security of these solutions.

Usernames and passwords are how most of us identify ourselves to almost every system around the world. For a long time now, security researchers have been looking for a better way. Traditional authentication just is not that safe or intuitive. Passwords can be stolen from a database or by a man-in- the-middle (MitM) attack, a keylogger, or even brute force; passwords could be exposed across the network; or multiple users could have knowledge of the password. Worse still, people end up having to remember a different password for each site they visit if they want to be safe, and often forget them. However, despite the emergence of new authentication tools such as certificates, smart cards or biometric systems, authentication systems based on passwords remain the most used due to their simplicity and low cost.

Various systems have been introduced to help make this process more secure, including two-factor authentication and third-party login services from Facebook, Twitter, Microsoft, and Google, notably. However, these systems also have flaws. Two-factor authentication can be annoying for users to deal with, while third-party login services rely on a third party, so if your Facebook account gets compromised, all of the sites you log in with that username will also be compromised.

Another problem of authentication systems based on passwords is the difficulty of providing a credentials revocation system that invalidates user credentials whenever necessary. To avoid non-authorized access, it is common to request a set of credentials to complete the authentication process, dividing the required credentials between different administrators. The presence of all the administrators or a subset of them is required to complete the authentication and access to system capabilities or the protected data. The utilization of two authentication factors enforces the security of the system, causing the attacker to access an element (certificate, token, smart card, mobile phone, etc.) and know a secret. The use of a Public Key Infrastructure (PKI) [1] allows checking the user permissions using certificates issued by a trusted third party called the Certification Authority (AC), while performing a query over the CLRs or using the OCSP protocol [2], the validity of a certificate can be verified.

In summary, traditional passwords present some problems related to the provided security, usability or difficulties to implement management operations which we aim to solve with the proposed authentication scheme. The weakness in password security can be solved combining two authentication factors and one-time password approaches. For this reason, we seek to design and implement a new password based authentication system to overcome the limitations of the traditional systems preserving the usability at a low cost. To fulfill this task, we have analyzed different cryptographic systems and how they can be applied to perform authentication operations using passwords in a simple way.

## 2. Related Works

One of the mentioned risks of using passwords is that the passwords can be stolen when they are introduced. Some schemes have been purposed to solve this problem. The periodic or after each use update of these passwords should enforce the system security but this method requires a bigger effort in maintenance and management tasks and could increase the risk of lost password. A solution that could simplify the maintenance tasks is the use of one-time passwords (OTP). OTPs are only valid during one authentication process, which prevents attacks due to the forwarding of network packets or a user could see a part or the complete value of the password. Some OTP systems, such as the Lamport scheme, uses a hash function to obtain a new password value from the last value after an authentication process occurs. Other schemes are based on time synchronization between the authentication machine and the client device, in such way as the password generation is performed using a cryptographic function that takes as input the current time and a shared password. Sometimes the time synchronization is replaced by counters showing the index of the current password inside a password sequence. There are many purposed schemes based in this approach and some of them are defined as standards, such as the systems S/KEY [3,4], OTP [5], HOTP [6] and TOTP [7]. These schemes use HMAC [8] functions with a shared secret key and counters or timestamps. Other approach consists in the authenticator request for a challenge to the user. This can be resolved only by a user knowing a secret predicate. Thus, returning a proper answer for the challenge, the user demonstrates the knowledge of the secret predicate without revealing information about it. These schemes are called Zero Acknowledgment Proofs. The exposed approaches require a service to obtains the current password from the shared key and the additional data to complete the authentication process. Due to the proliferation of mobile devices, such as mobile phones, tablets or laptops, this problem is not an insurmountable barrier. Tokens are another kind of mobile devices that allow performing these operations.

As we explained before, one way to provide credential revocation mechanism is using a PKI. Many public key algorithms such as RSA [9] or ElGamal [10,11] use modular arithmetic, in the same way as zero-knowledge proofs. The challenge presentation requires that the authentication device can read the challenge. There are authentication solutions that acquire information presented by the authenticator using QR codes such as the scheme proposed by Steve Gibson [12].

To implement the one-time password scheme, we used a zero-knowledge proof that presents a new problem; this is how the authentication device obtains the challenge. To enable communication between the device and the authentication device, we used QR codes. The entire solution involves the use of a PKI that allows implementing management operations such as revocation and expiration.

The main contributions of the proposed scheme are the zero-knowledge proof and the use of QR codes to perform the authentication operation. Although the proposed zero-knowledge proof is based in the scheme proposed by Guillou [13] published in 1988, many zero-knowledge proofs have been proposed after that. Many proposed schemes could be interesting to implement our system but most of them are based on modern cryptographic algorithms such as elliptic curves. This would make it difficult to integrate the zero-knowledge algorithm and a traditional PKIs but we considered the ideas exposed in papers by Chase [14], Ben-Sasson [15] and Cramer [16].

Some recent publications discuss the applicability of QR codes to authentication in mobile phones and some schemes have been proposed (e.g., [17,18]).

Finally, we studied the use of zero-knowledge proofs and multiparty secure computation. Some publications (e.g., [19]) are useful for us to design our proposed scheme and to identify various aspects which could improved in the future.

In the present paper, we present a new multifactor and distributed authentication system based on one-time passwords that aims to enforce the system security transparently to the system administrators. This document is structured as described above. First, in Fundamentals Section, the main technologies and tools used in the current project are described. In the next section, the mathematical concepts are demonstrated, and the required infrastructure is presented. Finally, the conclusions and the future work are described.

## 3. Fundamentals

In this section, the main technologies used by the distributed authentication system are presented, including the PAM libraries, JWT, QR codes, the zero-knowledge proofs and the Base 64 encoding system.

### 3.1. Pluggable Authentication Modules (PAM)

Linux-PAM is a collection of shared libraries, which allows the system administrator to configure what applications can authenticate users and the required procedures and credentials to complete the authentication process. The configuration files of every application that can authenticate users are located inside the /etc/pam.d. Every application using the PAM API has to define a name for the service, for example, login, and add its own configuration file. This file has to be the same as the service. For example, for the login application, the configuration file will be /etc/pam.d/login. This file will contain the required rules to complete the authentication process. The rules have the following format.

Type control module-path module-arguments.

The fields are separated by blank spaces. The first field, type, shows the operation type that has to be performed. The are four valid types:

- auth: This module verifies the user identity.
- account: This verifies the authenticated user has the required access permissions.
- password: This module allows to update the token associated to the user.
- session: This module allows implementing the required actions for the user before and after access to the service.

The control field has the responsibility to indicate to PAM when a module must fail. The valid values are:

- required: Returns an error after all modules are executed. The error is associated to the first module generated an error.
- requisite: An error is returned after the first module failure.
- sufficient: If a sufficient module succeeds, it is enough to satisfy the requirements of sufficient modules in that realm for use of the service, and modules below it that are also listed as sufficient are not invoked. If it fails, the operation fails unless a module is invoked after it succeeds. If a required module fails before a “sufficient” one succeeds, the operation will fail anyway, ignoring the status of any “sufficient” modules.
- optional: An optional module will only cause an operation to fail if it is the only module in the stack for that facility.

The module-path field shows the absolute path where the module is located or the relative path to the default location that will be /lib/security o /lib64/security depending of the architecture.

At the end, the module-arguments field is a list of tokens separated by blank spaces that allows configuring the module behavior.

The API PAM provides a message exchange system with the service that allows sending the data to be shown. The function used to present the data to the user is the conversation function. The PAM architecture must be extended for the current project. PAM provides the method misc_conv to implement the data presentation:

int misc_conv(int num_msg, const struct pam_message** msgm, struct pam_response** response, void* appdata_ptr)

This function does not provide any way to present a QR code. Thus, we have added a new method to the PAM kernel to provide a way to show the QR code and the corresponding conversation. This function has the same arguments and return type than the misc_conv function to maintain compatibility.

int qr_conv(int num_msg, const struct pam_message** msgm, struct pam_response** response, void* appdata_ptr)

This conversation function must be selected in the PAM configuration file for the module.

After that, a new PAM module has been implemented, named pam_multi_otp_unix.so. This new authentication module will generate a new challenge when it has requested a new authentication operation and will invoke the conversation function to present the challenge as a QR code and ask for the required authentication data. This module must be selected by the applications that desire using the distributed authentication method.

### 3.2. JWT

JSON Web Token (JSON) is a JSON-based open standard [20] for creating access token that assert some number of claims. The tokens are signed so they can be used to prove the identity of the source.

A JWT has three parts:

- Header: Used to identify the signing algorithm.
- Payload: Contains the claims to make.
- Signature: The signature of the JWT. The signature is generated obtaining at first the unsigned token as encodeBase64Url(header) + “.” + encodeBase64Url(payload). Then, the signature algorithm is applied over the unsigned token.

Thus, the final content of the JWT is:

token = encodeBase64Url(header) + “.” + encodeBase64Url(payload) + “.” + encodeBase64Url(signature).

In this project, a JWT is used to represent the challenge shown by the QR code. This JWT is called challenge JWT. The challenge JWT will contain the next claims as defined in the standards:

- Token Type (*typ*): Constant field set t “JWT”. This claim is placed at the header.
- Message authentication code algorithm (*alg*): The algorithm required to verify the signature. This claim is placed at the header.
- Issuer (*iss*): The identifier of the device asking for the password.
- Subject (*sub*): Constant field set to “challenge”.
- Audience (*aud*): The identifier of the user or group asking for authentication.
- Expiration time (*exp*): The expiration time of challenge defined in the current JWT set by the source using its clock.
- Issued at (*iat*): The time of challenge generation.
- JWT ID (*jti*): The identifier of the current authentication session.

The next custom claims are added to the payload:

- Challenge: The generated number for the challenge.
- Seed: The random number used to generate the challenge.

#### QR

A Quick Response Code (QR code) is an standard of data representation using a bidimensional point matrix. It was proposed in 1994 by the Japanese company Denso Wave and was standarized in June 2000. In December 2011, it was approved by the GS1 organization as standard for the automotive industry. The amount of information that can be represented by a QR code depends on the QR code type and the its size.

There are multiple QR code types:

- QR Code Mode 1 y Mode 2.
- Micro QR Code: This QR code only requires a one direction detection pattern. This allows printing it on a smaller surface. This QR can be used if the width of the image is module 2. The largest version of this code is M4 (17 × 17) and it can store about 35 characters.
- iQR Code.
- SQRC.
- Frame QR: This QR code contains a canvas area that can be used in various ways. Because this area can be used with characters and images, it can be used for multiple purposes such as authentication, promotions, etc.

In the current paper, a QR code is used to show the JWT containing the authentication challenge to allow it to be read by mobile devices. Because the JWT contains enough information to continue the authentication process, the QR representation mode does not need to provide an authentication method. A QR code with 101 × 101 and high error correction rate is used, which represents up to 3248 bits.

### 3.3. Zero-Knowledge Proofs

One of the main disadvantages of password based authentication protocols is the risk the password could be exposed. Zero-knowledge protocols emerge to solve this problem. A zero-knowledge proof is a cryptographic procedure executed between two parts, where one part, called prover *P*, tries to prove to the other part, called verifier *V*, it has knowledge about some sentence *s* without revealing the data it knows. This knowledge is called witness, *w*. Both parts know a predicate *R* that allows verifying *w* is a valid proof of *s*.

Different identification schemes based in zero-knowledge proofs have been proposed. Most of these algorithms use mathematical techniques similar to the techniques used by the public key cryptosystems. Designing a secure identification scheme is a complex task and different vulnerabilities have been detected in some proposed schemes, such as the Ong–Schnorr system [21,22]. There are schemes where the security is based in the difficulty of calculate the square root over modular rings. Feige [23] proposed in 1988 zero-knowledge based in this problem. Later Guillou and Quisquarter proposed a different variant [13] that is a generalization of the Fiat, Feige and Shamir protocol, which use roots with order greater than two.

### 3.4. Base 64

Base 64 [24] is an encoding system that uses 64 as encoding base. This is the largest base that can be shown only using printable characters. Most of the more popular variants use the characters A–Z, a–z, and 0–9 to represent the first 62 digits and the character used to represent the last two digits can be different. In our case, we use the character set A–Z, a–z, 0–9, +, and /.

## 4. Design and Implementation

The algorithms and software components conforming to the current solution are explained in this section.

### 4.1. Algorithm

In this section, the mathematical fundamentals of the current authentication system are exposed. Consider the device Authenticator (A), whose access is desired to manage the Authentication Manager (AM) that generates the initial data, the Authentication Service (AS) and the Authentication Client (AC) that is used by the administrators to obtain passwords. These components are described in Section 4.2. First, the value *id* is defined to identify the device whose access is managed. The next parameters are required to be configured after the *id* is set:

- Key length ($l_{k}$): The length in bits of the key pair generated for the current authenticator.
- Password length ($l_{p}$): The number of characters of the password requested by the authenticator.
- Number of users (*m*): Number of required users to complete authentication.

Once time the configuration of the required parameters has been completed, the Authenticator Manager generates the values required by the Authenticator.

First, two primes, *a* and *b*, are generated considering the configured length $l_{k}$. The prime number *a* is a strong prime. Thus, *a’* = *a*− 1 has a large prime factor denoted by *q*.

The integer *n* is obtained as

n=a·b

Thus, the Euler function of this value is obtained as

φ(n)=(a−1)·(b−1)

At same time, the value *g* is obtained, which is a generator of a subgroup with order *q* from the integers module *n*. The generator *g* exists because *q* is a prime factor of (*a* − 1) · (*b*− 1). The value of *q* must be large to avoid generating the same value in two authentication operations, as is explained below. The standard FIPS 186-4 recommends a length of 140 bits for *q* to generate RSA key pairs of 1024 bits. Thus, we think this length is large enough for our purposes. From the values *n*, *a*, and *b*, a key pair similar to RSA keys is generated, which is an integer *e* verifying the next expression is generated:

gcd(e,φ(n))=1

After that, an integer *d* is obtained such that

ed≡1modφ(n)

These values are stored by the Authentication Manager associated to the current Authenticator identified by its *id* value. The configuration parameters and the generated values are stored in a database which is accessible by the Authentication Service. The Authenticator receives the values [*d*, *e*, *a*, *b*, *g*], as explained in Section 4.2.

After the devices has been configured as Authenticator, *m* system administrators must be registered at least for the current device. An identifier is assigned to each user, denoted by $u_{i}$, and the group of authorized users denoted by *U* is defined as

$$U=u_{1},\ldots ,u_{m}$$

Each user must introduce a password in Base 64, whose length is $l_{p}$. Each of those passwords is denoted as $p_{i}$, for the user $u_{i}$, where i∈[1,m]. A transformation function is applied for every password obtaining a new integer $sk_{i}$, which it is the user final key. This function is implemented by a component that uses the PBKDF2 algorithm with HMAC-SHA1. The component is initialized with the parameters $l_{p}$ as desired key length and 10,000 as the required algorithm iterations. This value has been chosen considering the recommendations in NIST guidelines published in June 2017. The generation function receives two parameters, a randomly generated initialization vector and the user password.

$$sk_{i}=generate\left(p_{i},iv_{i}\right)$$

The value of the initialization vector $iv_{i}$ is selected such that $sk_{i}$ verifies

$$gcd(sk_{i},\varphi \left(n\right))=1$$

Thus, a value $e_{i}$ could be obtained for each user such that

$$e_{i}\equiv e\cdot sk_{i}^{-1}mod\varphi \left(n\right)$$

So

$$e_{i}\cdot sk_{i}\equiv e\cdot sk_{i}^{-1}\cdot sk_{i}\equiv emod\varphi \left(n\right)$$

Once time these values have been generated, the corresponding value $e_{i}$ is stored for each user. All system administrators should load in their own AC the values $\left[iv_{i},n\right]$.

The following steps are taken to perform the authentication process. When the command login is executed in the device A, the PAM module of this machine executes the following operations. A random password *p* with length $l_{p}$ is generated. Then, a random integer *r* is generated and the key *k* of A is obtained as follows

$$k\equiv r^{-1}{\left[p\right]}^{\left(1/d\right)}\equiv r^{-1}{\left[p\right]}^{e}modn↔p\equiv {\left(rk\right)}^{d}modn$$

A shows the password *p* and an access counter *j*, composing the challenge. No information about the value of *k* from the value *p* if the value *e* is not known can be obtained.

Each administrator receives these values and, introducing the corresponding password $p_{i}$, recovers the key $sk_{i}$ from the stored initialization vector $iv_{i}$. AC generates a random value $u_{i}$ with length $l_{p}$ bits and calculates

$$x_{i}\equiv {\left(u_{i}p\right)}^{s_{i}}modn$$

After the AC is authenticated in AS using its certificate, the system administrator sends to AS a request with the values $\left[x_{i},j\right]$ and AS returns the value $y_{i}$ calculated as follows:

$$y_{i}\equiv g^{j}x_{i}^{e_{i}}\equiv g^{j}{\left(u_{i}p\right)}^{e}\equiv g^{j}u_{i}^{e}rkmodn$$

The system administrator applies a mask to the value $y_{i}$ so that it obtains a value with length $l_{p}$ bits denoted by $m_{i}$. Then, the system administrator introduces the values [$m_{i},u_{i}$] in the A. Now, the authenticator knows the values [*g*, *j*, *d*, *e*, *r*, *k*]. Thus, the authenticator can calculate

$$y_{i}^{\prime }\equiv rkg^{j}{u_{i}}^{e}modn$$

If A applies the same mask as the AC to the value $y_{i}^{\prime }$, it obtains $m_{i}^{\prime }$. If the equality $m_{i}^{\prime }=m_{i}$ is verified, then the identity of the user has been proven. The authentication process requires the knowledge of data only known by the administrator and data stored by the AS that checks the administrator permissions. A valid client certificate is required to access the AS. This aspect allows implementing revocation operation for users, because revoking the client certificate the user cannot access the AS. This capability can be easily implemented using operations commonly available in most of the existing PKIs.

### 4.2. Solution Architecture

Several components are required to implement an authentication based on the algorithm presented in the last section. In the current section, we describe the components of the system and their connections. The distributed authentication system is composed of several components that can be classified in three categories:

- Workstations: The devices whose access control is managed using the distribution managed system. A workstation that has been registered is called Authenticator (A).
- Client components: The devices hosting a client application used by the workstation administrators to solve authentication challenges and generate passwords. They connect to the server components to complete the authentication operation. The device is called Authentication Device (AD) and the application is called Authentication Client (AC).
- Server components: The server components provide services to register new clients and workstations and to verify client permissions. The authentication services must be accessible by the client devices.

Since this solution uses digital certificates to perform some operations, a Public Key Infrastructure (PKI) must be available. A PKI is a set of roles, policies, and procedures needed to create, manage, distribute, use, store, and revoke digital certificates and manage public key encryption. It is composed of several applications that offer different capabilities required by the described authentication system:

- Certification Authority (CA): Issues, signs, stores and manages digital certificates for the requesting entities.
- Registration Authority (RA): Verifies the identity of entities requesting digital certificates. The RA application requests to CA to issue a new digital certificate.
- Validation Authority (VA): Provides services to verify the status of digital certificates. A digital certificate can be revoked, suspended or expired. Thus, in those cases, the digital certificated is not valid and it cannot be used to perform public key operations.

**Figure 1:**
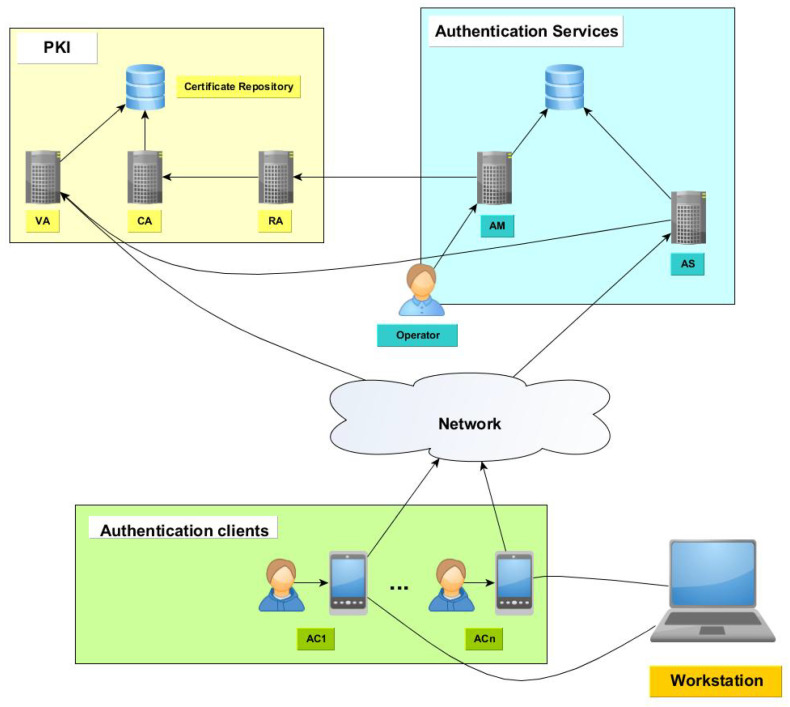
Distributed authentication system architecture.

The relationships among the mentioned components are shown in Figure 1. Once the implemented and required components have been exposed, we describe the components implemented to build the authentication system.

The server side is divided in two components: the Authentication Manager (AM) and the Authentication Service (AS). The AM provides a web interface that is locally accessible by a set of system operators (Authentication Operator, AO). This component has two main responsibilities:

- Register the workstations that use the proposed authentication solution in the system.
- Register system administrators for a given workstation.

**Figure 2:**
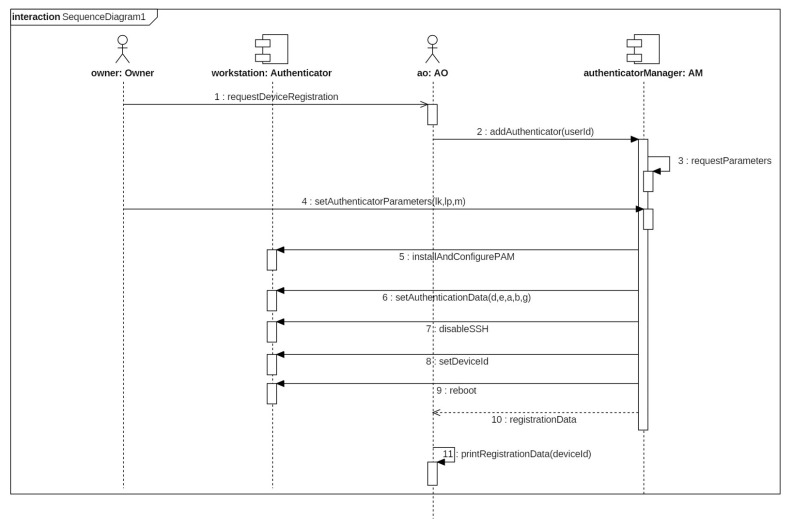
Sequence diagram for authenticator.

To add a new workstation, the owner of that device requests to the AO to register the device in the system. The AO verifies whether the owner is authorized to register device and then initializes the workstation. The device must be accessible from the AM and have an SSH server enabled. The AO introduces in the system the authentication parameters [$l_{k}$, $l_{p}$, *m*] and, then, the AM connects to the workstation to install and configure PAM. After that, it installs the authentication data [*d*, *e*, *a*, *b*, *g*] described in the last section. The AM registers the new authenticator in the system and, finally, it disables SSH server and reboots the workstation. The workstation is now configured as authenticator. This process is shown in Figure 2.

**Figure 3:**
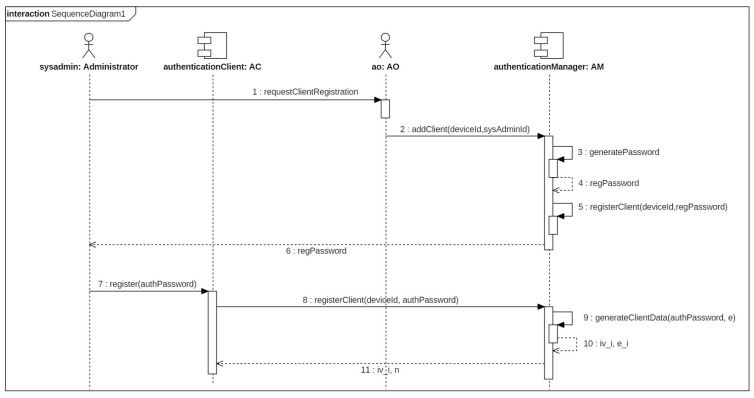
Sequence diagram for AC.

Once the authenticator has been registered, the authenticator administrators is associated to that workstation by the AO. The AO requests to the administrators their identities and passwords. Then, the AM connects to the RA to issue one client certificate for each administrator and generates a password to download the certificate and the client authentication data. Finally, the system administrators initializes their ACs in their ADs using the generated password. The procedure is shown in Figure 3.

**Figure 4:**
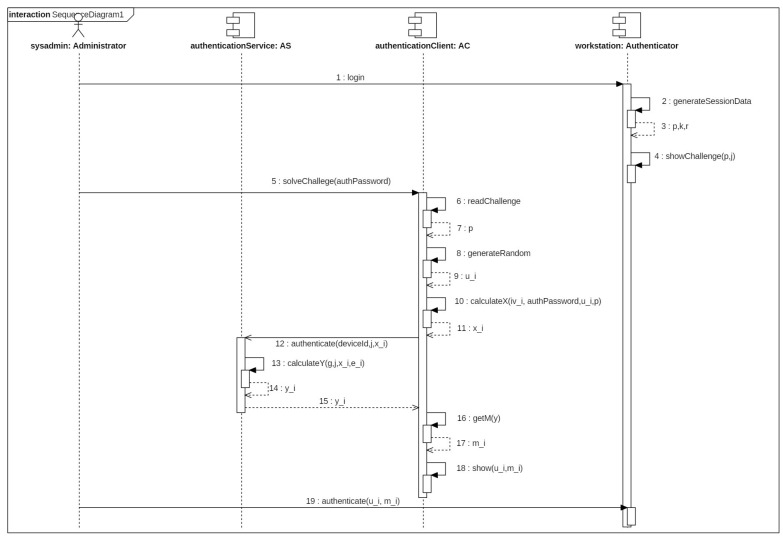
Sequence diagram for authentication operation.

A service cluster called Authentication Server (AS) is available to perform authentication operation. The service AS allows solving the authentication challenge posed by the authenticator device A to the authorized users. The user uses the AC application where the client certificate of that should be loaded because the certificate is necessary to establish a secure connection with the service AS. The AC application reads the challenge data presented by the authenticator as QR code after the user has authenticated into the AC with the private password. Then, the AC application calculates an intermediate value, establishes a secure connection with the service AS and sends it the obtained value. The service AS returns a new value that is processed by application AC to calculate the authentication token. The final token is shown in the mobile device to the system administrator who introduces the value into teh login console of the authenticator device A. This operation is shown in Figure 4.

### 4.3. Security Aspects

The algorithm security relies in the difficult to solve the problem of prime factorization of integer numbers. Find the factorization of the number *n*, for an integer enough large, it is not possible in a polynomic time for any known algorithm. The recommended minimum length is currently 2048 bits, but many authors recommend 4096 bits. However, much current hardware, such as smart cards, HSMs, etc., does not support this key length. Because this kind of algorithms offers only computational security, depending on computing time, the required key length of *n* could increase with the time. This variation would depend on the improvement of device performance and find more efficient algorithms. Thus, when choosing the key length, the duration of the keys offered should be considered. At present, NIST speculates that 2048 bits keys would be valid until 2030.

Continuing with the algorithm analysis, the authenticator device shows the value *p* and the counter *n*. To obtain some information about *k* and *r*, knowing the value of *e* should be needed. Even knowing the value *d* and *n*, it is not possible to obtain *k* and *r* if the prime factorization of *n* is not known.

Related to the value of *g*, if an attacker could impersonate the system administrator, he could not obtain information about the value of *g*, provided that counters such as 3≤j<φ(n) would be used due to obtain *g*, which requires knowning the prime factorization of *n*.

## 5. Conclusions

The current architecture allows deploying the system in a local network or in a remote network accessible through Internet. The prototype implementation uses a simple PKI implemented for the current project. The client and server applications access PKI API through its REST API. In the future, the applications must verify certificate status using the standard OCSP protocol or accessing the CRLs. A standard method to access RA services must be defined. This is the main problem to connect to different PKIs because it requires the RA provides an API to access its services remotely.

The proposed scheme preserves the usability of the traditional password based schemes adding as the only requirement access to a device connected to the Internet with the Authentication Client application installed. Due to the proliferation of mobile devices nowadays, this is not a difficult problem to solve. The most difficult requirement is ensuring the Internet connection when the authentication process is going to be performed. Thus, an interesting improvement would be providing a new operation mode allowing a preload step that completing the authentication process without connectivity. To implement this capability, we are evaluating the utilization of some timestamp systems. This scheme provides the security of a system using two authentication factors, the possession of a client certificate and the knowledge of a password enforced by the use of one-time password scheme. These capabilities enforce the security of the traditional password based schemes. The password distribution across multiple parts help avoid the malicious use of software services, requiring the presence of a minimum group of administrators to accomplish every task. We will evaluate the possibility of integrating new authentication methods such as coordinates cards to enforce the system security in future work.

Finally, we will study the applicability of the Shamir secret sharing scheme. Since the component AS is required to verify the administrators permissions, we will evaluate the advantages of calculating an unique password for the authentication process. This password is calculated by the AS from data received from the administrators and it is introduced into the login console of A.

## Abbreviations

The following abbreviations are used in this manuscript:

AM	Authentication Manager
AS	Authentication Service
AO	Authentication Operator
AC	Authentication Client
AD	Authentication Device
A	Authenticator
CA	Certification Authorty
RA	Registration Authority
VA	Validation Authority
PKI	Public Key Infrastructure
OTP	One Time Password
MITM	Man in the Middle
HMAC	Hash based Message Authentication Code
CRL	Certificate Revocation List
OCSP	Online Certificate Status Protocol
